# Impacts of maternal mortality on living children and families: A qualitative study from Butajira, Ethiopia

**DOI:** 10.1186/1742-4755-12-S1-S6

**Published:** 2015-05-06

**Authors:** Mitike Molla, Israel Mitiku, Alemayehu Worku, Alicia Ely Yamin

**Affiliations:** 1 School of Public Health, College of Health Sciences, Addis Ababa University, Addis Ababa, Ethiopia; 2 Department of Public Health, College of Medicine and Health Sciences, Wollo University, South Wollo, Dessie, Ethiopia; 3 Global Health and Population, Harvard School of Public Health, Boston, Massachusetts, USA; 4 Franςois-Xavier Bagnoud Center for Health and Human Rights, Harvard University, Boston, Massachusetts, USA

**Keywords:** Ethiopia, maternal mortality, children, families, health, nutrition, education

## Abstract

**Background:**

The consequences of maternal mortality on orphaned children and the family members who support them are dramatic, especially in countries that have high maternal mortality like Ethiopia. As part of a four country, mixed-methods study (Ethiopia, Malawi, South Africa, and Tanzania) qualitative data were collected in Butajira, Ethiopia with the aim of exploring the far reaching consequences of maternal deaths on families and children.

**Methods:**

We conducted interviews with 28 adult family members of women who died from maternal causes, as well as 13 stakeholders (government officials, civil society, and a UN agency); and held 10 focus group discussions with 87 community members. Data were analyzed using NVivo10 software for qualitative analysis.

**Results:**

We found that newborns and children whose mothers died from maternal causes face nutrition deficits, and are less likely to access needed health care than children with living mothers. Older children drop out of school to care for younger siblings and contribute to household and farm labor which may be beyond their capacity and age, and often choose migration in search of better opportunities. Family fragmentation is common following maternal death, leading to tenuous relationships within a household with the births and prioritization of additional children further stretching limited financial resources. Currently, there is no formal standardized support system for families caring for vulnerable children in Ethiopia.

**Conclusions:**

Impacts of maternal mortality on children are far-reaching and have the potential to last into adulthood. Coordinated, multi-sectorial efforts towards mitigating the impacts on children and families following a maternal death are lacking. In order to prevent impacts on children and families, efforts targeting maternal mortality must address inequalities in access to care at the community, facility, and policy levels.

## Background

Although Ethiopia has advocated for all mothers to give birth in a health facility in an effort to combat high maternal mortality rates [1], the maternal mortality ratio (MMR) in Ethiopia remains one of the highest in sub-Saharan Africa. Furthermore, the Ethiopian Demographic and Health Survey (EDHS) has indicated no marked decline in recent years, reporting an MMR of 673 maternal deaths per 100,000 live births in 2005 and virtually the same rate, 676 maternal deaths per 100,000 live births, in 2011 [2,3]. Ethiopia is one of the least urbanized countries in the world, with only 16% of the total population (estimated in 2012 at 91.7 million) living in urban areas, posing considerable access issues on the majority of the population [3,4]. Related disparities persist between rural and urban populations – for example, the likelihood of urban women giving birth in a health facility was 4.6 times that of rural women in 2011, signifying only a slightly lower discrepancy than in 2000 (4.9) [5]. Similarly, Ethiopia has a total fertility rate (TFR) of 4.8 children per woman, yet the TFR in rural areas is nearly three children per woman higher than in urban areas [3]. In the Southern Nations, Nationalities and Peoples region of Ethiopia, in which 90% of the population lives in a rural area, only 14 of the 184 health facilities perform all seven basic signal functions of Emergency Obstetric and Newborn Care (EmONC) and closer proximity to these care facilities has been linked to lower estimates of neonatal mortality [6]. Moreover, while the under-five mortality rate in Ethiopia has declined by two-thirds, achieving Millennium Development Goal (MDG) 4, neonatal mortality which comprises roughly 50% of all infant deaths in Ethiopia, remains high at 37 deaths per 1,000 live births without significant decline in the past ten years [2,3,7].

It is well documented that infant and under five survival is highly correlated with child nutrition and other important child health care practices attributable to maternal involvement and prioritization of child health [8-10]. For example, early and exclusive breastfeeding, a nutritional advantage only a mother can provide, has been shown to substantially lower the risk of neonatal death [8]. Routine immunization, another critical health care practice which mothers prioritize, has similarly been associated with a reduced risk of infant mortality [10]. Associations between maternal death and long term child health and survival however, are less understood. A growing body of literature is exploring the catastrophic effects of maternal death, loss of the primary caregiver, on older children in addition to newborns and infants [11-16]. The costs of maternal deaths on living children, family and other dependents are widespread, impacting future generations of individuals as well as overall wellbeing and economic progress at the country level. To this effect, evidence has shown that investing in the prevention of maternal mortality through the prioritization of the education, empowerment, and sexual and reproductive health and rights of women and girls is a cost effective intervention [17], saving the lives and promoting the livelihoods of mothers, children, and families [18].

Though the magnitude of maternal death in Ethiopia has been documented [2-4,7], the true toll of maternal mortality is under-valued; and long-term impacts on children, families, and communities have yet to be adequately explored. In order to contribute to the growing body of literature aimed at documenting these impacts, a four country, mixed methods study including South Africa, Malawi, Tanzania, and Ethiopia, was undertaken. This paper presents qualitative findings from the Butajira, Ethiopia study site; and demonstrates the extensive impact of maternal mortality on orphaned children.

## Methodology

### Study site, design and participants

The data presented here documents the impact of maternal deaths on living children in one of the oldest Demographic Surveillance System (DSS) sites, Butajira Rural Health Program in Butajira, Ethiopia. Butajira is a rural district situated in the former Meskan and Marko districts, in the Southern Nations, Nationalities, and Peoples’ Regional State (SNNPR), approximately 130 kilometers from Addis Ababa. The Butajira Rural Health Program (BRHP) was initiated in 1986 to collect longitudinal demographic and health data and covers a population of 74,407 individuals comprising 12,407 households [19] within nine rural and one urban kebeles (villages) [20]. This study employed in-depth qualitative research methods in five kebeles (villages) serviced by the BRHP: Bati, Dirama, Misrak Meskan, Dobena, and Butajira town K04. Nine health posts (one per rural kebele) provide most primary and preventive (including maternal and child health care) health services to the population, though three health centers and two hospitals (a zonal hospital and an NGO hospital) also service the area. Along with this qualitative study, results from a quantitative analysis on the impact of maternal mortality, using twenty-five years of BRHP DSS data, have been published elsewhere [21].

The inclusion criterion was defined as the death of a woman during pregnancy, childbirth, or 42 days following the termination of the pregnancy, as per WHO standards [14]. Data collection occurred between August and October 2013. Patient records from Butajira Zonal Hospital were used to identify women who had died from maternal causes. Snowball sampling was then used to identify family members of these women. Among the 28 maternal deaths captured, only one woman was divorced while 5 were in polygamous marriages and the rest were in monogamous marriages. Most women (n=15) had either primary or secondary education, and (n=15) delivered at home, Fourteen women were aged between 30-39, and nine left behind 3-5 children (Table 1). Topics covered in interviews with family members included: (1) general socioeconomic characteristics of the family; (2) drivers of maternal mortality and the impacts on the children and family; and (3) availability and accessibility of services for maternal orphans. If there was more than one child orphaned due to a maternal death, the impacts of the mother’s death on each child were discussed.

**Table 1:** Profile of women who died of maternal causes (n=28), Butajira, Ethiopia

Variable	Number
Type of informant	

Husband Mother Mother Father in law Brother/sister Nephew/ son	14 3 3 6 2

Age group	

17-19 20-29 3-39 40-49 No data	4 3 14 3 4

Marital status	

Married (monogamous) Married Polygamous Divorced	22 5 1

Place of delivery	

Home Hospital No data	15 12 1

Education	

Illiterate Primary 1-6 Secondary 7-12 Tertiary 12+ No data No data	10 9 6 1 2

ANC follow up	

Yes No No data	13 5 10

Number of children left behind	

First time pregnant 1-2 3-5 6-8 ND	2 8 9 4 4

Two sex-stratified Focus Group Discussions (FGD) were conducted in each of five kebeles (four rural and one urban kebele), totaling 87 participants. Roughly eight people participated in each FGD. FGD participants were identified using purposive sampling, and included teachers, traditional birth attendants, religious leaders, community health workers (health extension leaders), community leaders, farmers, and other community members. Topics covered in the FGDs included differences between families who were and were not affected by maternal mortality, impact of maternal deaths on living children from infancy through adolescence, and resilience.

Additionally, thirteen stakeholder interviews were conducted focusing on policies and programs available for maternal orphans, and challenges for implementing such programs and policies, such as financial issues and political will.

### Data collection

All data collection tools were prepared by the Harvard School of Public Health in English and translated into Amharic by a professional translator. The interviews were conducted by four lecturers from Addis Ababa University. All in-depth interviews and FGDs were conducted in a place selected by participants that provided optimum privacy.

Each in-depth interview and focus group took between 1.5 and two hours to complete. Family member and focus group participants also received 100 Ethiopian birr (*$*5.00 USD) for the time spent. All interviews and focus group discussions were transcribed from the digital recording and translated into English by an experienced professional.

### Data Analysis and interpretation

All interviews were imported and coded using NVivo 10 qualitative analysis software [22]. The interviews were coded independently using an inductive approach by two members of the research team and discussed for differences and emerging codes. The findings were analyzed using phenomenological approach where meanings were assigned as per the informants’ representation of the phenomenon. Salient quotes were used to express the experiences and perceptions of the informants.

### Ethics statement

Study protocols were approved by the Harvard School of Public Health Institutional Review Board and by the Addis Ababa University, College of Health Sciences Institutional Review Board. Informed consent was read aloud verbatim by the research assistant or interviewers and all participants consented through either a signature or thumb print.

## Results

### Household changes: caregiving and poverty

Unlike in Malawi, South Africa, or Tanzania [13,15,16], we were not informed of any formal procedures regarding the care or placement of children with family members following a maternal death in Butajira. However, according to study participants, fathers rarely take on child care responsibilities following the death of a wife. In Butajira, common roles for women include providing child care, caring for the home, cooking, and generating supplemental income by selling handicrafts or agricultural products from their gardens, while men primarily carry the responsibility of earning income in farming. This gendered division of household duties leaves fathers unprepared to assume caregiving responsibilities and fulfill the needs of the family without support following a maternal death. Husbands reported that their capacity in handling their families was halved because of their wives’ deaths. As one informant described:

..I am hurt, I had sold my land and I am renting the rest, I only have this land am still in grief. My late wife used to support me by taking care of the household chores and also she used to sell ‘khat’ and cabbage…

The effects of losing any supplemental income generated by their wives, compounded by extensive funeral costs and time spent away from work for funeral ceremonies, often transformed a family’s economic outlook. Funeral costs rapidly deplete a family’s available resources and force households to utilize reserves that have been saved up over time; or sell valuable, often income-earning assets, leaving families in substantially lower economic positions than they were prior to the maternal death [23,24]. As this informant shared,

It [the reason why I stopped farming and am now renting my land] is because of the condition of our life and the weather conditions were not good, so, the production became less. And when we became short [financially] to afford the necessary things [I rented the land out]. But before her death we used to support each other to fulfill our needs. Life is not as easy when you lead it alone as when you have a partner.

Half of the family members interviewed described situations in which families were given support by family on the maternal or paternal side, including taking childcare responsibilities on completely, or providing housing, cooking, material goods such as clothing or school exercise books, and money; and eight described situations in which financial and/or emotional support was offered by the surrounding community. This support, while often critical to the health and well-being of the family suffering a loss, diminishes the supporting family’s capacity to meet their own needs. As this grandmother supporting her son’s infant child explained, “I used to work in the market, but I cannot go out of the house now as I have to take care of the newborn. One of my daughters had also dropped out of school to help me...”

Re-marriage following a maternal death came up in many interviews as a contested issue. While re-marriage is seen as an option for offsetting the burden of child and household care responsibilities that fathers and supporting family and community members feel following a mother’s death, participants explained that, in practice, bringing a new wife into the home often leads to family disputes regarding the division household labor, both between children and their stepmothers and between husbands and their new wives. Some participants perceived stepmothers as likely to mistreat orphaned children, especially if they have biological children of their own to care for. Only three participants relayed positive experiences or opinions regarding relationships with stepmothers (i.e. improvements in the care of orphans following a re-marriage), whereas fifteen shared negative opinions, or personal experiences of stepmothers refusing to provide care to children (i.e. cooking for them), or, more often, of frequent arguments at home between stepmothers and step children. As this father explained,

she [stepmother] was good, [but] her behavior has changed since she had her own kid... the children [maternal orphans] argue with her most of the time, when I [father] try to mediate, they say she is not fair, she gives more food for her children than us... I am planning to send the older one to Addis, he may lead a better life there...

As noted, girl children are often forced to fill their mother’s role in the home following a maternal death, even at a young age and at the expense of an education, because fathers do not tend to take on what are considered female responsibilities following a maternal death. One father explained that, because he had to attend to farming and there was no one to “look after the house” following his wife’s death, his daughter had to drop out of school to take on those responsibilities, and got married three years later, never returning to her studies. One focus group participant described the household responsibilities that girl children take on as follows:

It’s obvious, if she doesn’t have a mother it’s she who will do everything, everything that her mother used to do. Males, they don’t spend much time at home and they expect a lot from her. It’s her who will discuss with the father on the subject of buying something for the house, boiling coffe etc. She is responsible for everything…if the daughter in the family is old enough to do some house works, she will be the one in charge: she will clean the house, clothes, dishes etc.

We also were informed on multiple occasions of older daughters dropping out of school in order to work and contribute to the family’s income. While this occurrence is not unique to families who have lost a mother, participants felt that the impacts of these situations on children were worsened when they did not have a mother versus a father.

I know of man with similar cases for instance. The mother is not alive. So, the father is raising the children and the older daughters are working as maids in this town. And when we compare families who lost the mother with families who lost the father the one who lost the mother suffers more than the other one.

### Impact on neonatal survival

While fourteen infants were born alive to the 28 women who died from maternal causes, only 3 survived to 6 months. According to key informants and FGD participants, causes of neonatal deaths in the community were mainly lack of proper feeding, especially breast feeding, due to lack of a mother. Most family members interviewed indicated that formula was unaffordable, and while health facilities provided the feeding supplement “plumpy’ nut” for free, caretakers reported using it for primary rather than supplemental feeding, and observed that, as such, it had a limited effect on child survival. Participants uniformly expressed that the growth and nutrition of children who were breastfed and cared for by their mothers was exponentially greater than that of orphaned children:

But in general, whatever kind of good treatment he is receiving, a kid who lost his mom and a kid who lives with his mom cannot be the same. If his mother is alive, the kid could grow with health, receiving simply his mom’s breath, breast feeding and her warmth.

Participants reported using a mixture of infant feeding types, such as cow and goat’s milk, powdered milk, formula, and community members’ breast milk, to feed maternal orphans. Six participants mentioned foods not suitable for infants, such as cabbage, tea, and traditional dishes, such as “mitin”, which is comprised of sorghum, peas, teff flour and barley. Interestingly, while participants generally felt that breastfeeding maternal orphans by grandmothers, female relatives, or other breastfeeding women in the community was a common and beneficial practice, a few felt that using “mixed” breast milk to feed infants was harmful to infants, causing ill health. One family member specifically noted the danger of transmitting disease via breast milk, while others attributed poor infant health generally to being fed by multiple women:

The reason why his body has swollen was because he breast fed by numerous women. Had he got his mother's breast milk that would have not been the case. He was [breast-fed] by many women just because they wanted to save his life, and he was not felt well anyway, being breast-fed by numerous women is not good/healthy.

### Health and health care seeking

In addition to the financial and household related burdens felt by a family following a maternal death, stakeholders, family members, and focus group participants described differences in child health and health care seeking for families who have and have not experienced a maternal death:

No one will bother and take a child to health institution at exact time for immunization. So its day for immunization will be forgotten and pass unless a mother is alive. A mother will wash, feed and immunize her child [in a] timely [manner] which couldn’t be true for any other person. Me myself if I had a child I will never leave him for a moment and give all necessary care every time but if I raise other’s child I will not bother as I do for my child

As the focus group participant cited above notes, even step-mothers and female care givers who step in to help a family following a maternal death will not prioritize the health of maternal orphans the same way they will for their own children. Several male care-givers (fathers and family members) explained that they often delay seeking health care for their children, hoping that they will get better on their own and therefore will not need costly medical treatments, which become more challenging to afford due to the loss of income generated and funeral ceremony costs incurred following a maternal death. As one father explained:

A: When his case worsens, I take him to the hospital, these days, it is better

Q: So you spent a lot for medical fees?

A: Yes a lot

Q: Did you have to sell anything to take the baby to the hospital?

A: Yes

Q: What did you sell, for example to provide milk etc…?

A: Yes, we had an ox; we sold it

Moreover, prior negative experiences at the facility level—such as perceived inadequate care and multiple transfers—as well as costs incurred paying for services and traveling to and from facilities, led to further hesitations to seek care at the community level. As one stakeholder explained, “To tell the truth, there is nobody who hates comfort, it is because of financial problem. It is those who have financial problem that do not want to give birth at health facility”.

Though poverty affects the majority of families in the study area, and while women are often unable to access maternal health care due to intersecting barriers of poverty, distance to the health facility, and lack of decision making power, participants consistently explained that when mothers are alive, they make every effort to get their children the health care that they need, even if they are not the primary decision makers for the family, as they often are not. As this key informant stated,

A mother is everything for a family. Father may think for his family but not as mother does; when a child gets sick she will notice and take[him or her] to the health institution or raise the idea to take [him or her] as prompt as possible with the father

As reflected in this and other emerging themes related to migration and education of orphan children, when children lose their mother, they lose their primary advocate.

### Impacts on older children: education, early marriage, and migration

As previously described, girl children were more likely to drop out of school following the death of a mother, given cultural gender norms regarding men and women’s roles. However, participants described additional circumstances that commonly necessitate school drop out for boy and girl children. For example, though school is free in rural Ethiopia, educational costs related to clothing, exercise books, pens, etc. prevented children from attending school. While older girl children dropped out to care for younger siblings and take care of the house, boy children were required to help their fathers or caretakers in manual labor, such as farming, which prevented them from studying and eventually led to drop out. As this family member described:

Had their mother been alive, for example, they would not be going to look after the cattle, also they would have been studying after they came back from school, they could get a better education too. They are not learning as they are supposed to because of her absence.

Again the theme of a mother’s love and guidance permeated discussions of education. Participants explained that, without a mother to provide encouragement, or to prioritize children’s nutrition at home, children lost motivation to attend school:

They come back from school and can’t find food because their mother isn’t around. They can’t learn [and are] hungry and thirsty and their morale is reduced, I think…He could be a bright student but his mother is not there to tell him to study etc…For example, I am ignorant but I come from an educated family, my mother loved it when her children studied, and she managed to do some trade and send them to school…

Migration to work in major cities in the region or in Middle Eastern countries was another common outcome for children following a maternal death, for girls and boys. In interviews with family members, we heard of nine children who migrated out of Butajira for work; 3 of whom were male. Though specific jobs were not always disclosed, working in food service (boys) or as a house maid (girls) was reportedly common. Participants described situations in which children asked to be sent by their families to work in the Middle East, in order to make money and/or leave tenuous relationships with step-mothers behind; as well as situations in which guardians made the decision to send their children, because they couldn’t afford to send them to school, or felt that working abroad offered them the best financial outlook. Though migration for work is a common occurrence amongst poor youth in the region, participants shared that losing a mother greatly contributed to a child’s likelihood of migration: “had her mother been alive she could have been enjoying her mother’s love here… also she wouldn’t have gone abroad”.

### Available support

Our findings indicated that there was no structured support system for maternal orphans. Stakeholder informants described that, while various NGO’s offer support systems for children, they are mostly directed to orphans from HIV, and are neither well organized nor systematized, as different NGOs cover different geographic areas and take different approaches to their programming. A stakeholder from one of the Ministries indicated the lack of systematic response as follows, "Though the country has adopted a child protection policy, it is poorly implemented and [there is] no coordination among sectors"

Child health and social protection programs and policies do not overlap; as this stakeholder went on to explain. While health extension workers have the capacity to conduct household assessments following a maternal death, they face competing service delivery priorities and which limit their involvement with communities. Coordinated efforts between health facilities, NGOs, Ministries, and communities, cutting across health and social welfare are needed to prevent maternal orphans from slipping through the cracks. In addition to this, however, several stakeholders also mentioned the need to improve quality at health facilities, to prevent maternal deaths in the first place. According to one stakeholder at the facility level:

What I want to say is that, the government policy is good, but the nurses and midwifes also other professionals have to get additional trainings in order to made them fit. Right now there is a problem of quality, but not quantity; a lot of things have to be done by the midwifes and by the nurses, in order not to make mothers die, we have to be able to refer the mothers immediately after detecting the risks when they are on anti-natal care and this is done in every health facility so, I believe that there is a quality problem on the professionals.

## Discussion

This study has highlighted the burden which families and children face following a maternal death. Without a mother, newborns and children face nutrition deficits, impacting their survival and growth; and older children drop out of school to care for younger siblings and contribute to household and farm labor which is often beyond their capacity and age, and often choose early marriage or migration in search of better opportunities. Family fragmentation is common following maternal death, leading to tenuous relationships within a household with the births and prioritization of additional children further stretching limited financial resources.

Our finding regarding sub-optimal infant feeding practices among maternal orphans supports existing literature from low resource settings [8,11,25]. Moreover, a recent community-based nested case control study in rural east Ethiopia found that children whose parents did not jointly decide on the treatment of a sick child, mothers’ lack of facility access, and non-exclusive breast-feeding were significantly more likely to experience wasting [26]. Considering that maternal mortality exacerbates each of the aforementioned predictors, it is reasonable to hypothesize that maternal orphans face worse odds of malnutrition and wasting.

Underpinning all themes uncovered in our analysis was the perception of mothers as the primary nurturers of and advocates for their children. Participants continually stated that the lives and opportunities for their children would have been greatly improved had their mothers lived. These sentiments compliment research that has begun to more rigorously investigate the role that women’s agency plays in child health. A case-control study in Butajira found low decision making capacity of women to be associated with three fold greater odds of under-five mortality in adjusted analyses [27]. Similarly, a recent study on EmONC access in the SNNP region found differences in maternal autonomy to contribute to overall and socio-economic inequalities in early neonatal mortality [6].

We found that, following a maternal death, children may be separated, placed with various relatives, or remain under the care of their father, and sometimes, step-mother. Where to place children appears to be a complicated question, depending upon multiple factors such as family size and connectedness, economic status, distance, father’s marital status, and the ages of the children left behind. Research from the Amhara and Oromia regions of Ethiopia suggests that newborns are not valued until the 40^th^ day of life [28], which may be related to the lack of standard or formal decision making processes regarding the placement of children, as well as to our finding of heightened neonatal mortality following a maternal death. We also saw that men, as a result of defined traditional gender roles, are generally unable to assume household responsibilities in the absence of their wives as they have never been involved in chores or childcare and are consequently ill-equipped to incorporate these tasks into their daily routines. As a result, orphaned girls are the first line victims who drop out of school to substitute for their mothers in caring for their younger siblings and tending to household chores. Other studies similarly showed that young girls in the region, including orphans, are often forced to prioritize household labor and childcare over education, impacting future economic opportunities [13,29,30]. Though labor migration is not specific to maternal orphans, key informants indicated that migration was often viewed as choice, made by girls and/or their families to escape the overwhelming household responsibilities put on them following a maternal death. A study conducted to assess female international labor migration in Ethiopia also cites the unfair division of domestic responsibilities and traditional gender roles as contributing factors for girls dropping out of school early and subsequently traveling to the Middle East to find work in the informal labor market [29]. While the ability to travel to Arab countries to work is often perceived as an advantage because these countries are much wealthier than Ethiopia and hold the promise of well-paid opportunities, the majority of the girls and women who migrate to the Middle East do so through illegal channels and are exploited and abused upon reaching their destination [29]. This reality is unknown to many Ethiopian families who are misinformed and view this as a chance for girls and women to earn their own living and provide money to their families, rather than a source of family fragmentation [29].

This study is not without limitations. Qualitative study findings are not generalizable outside of the study population and our small sample size and study design eliminate our ability to examine questions of causality. However, these findings contribute to a growing literature that is documenting the impacts of maternal mortality in developing countries. This is the first study of its kind in Ethiopia; and study findings are consistent with other emerging evidence [12-14,16,21,31], solidifying their importance in shaping future development priorities after the millennium development goals.

## Conclusions

In addition to the need to improve services aimed at mitigating impacts of maternal death on children, such as enhancing systemic linkages between health and social welfare programs, and strengthening social protection schemes to better address the needs of vulnerable children and families, our findings support the need to redouble efforts to combat maternal deaths, which are the root cause of the impacts on families and children documented here. Maternal mortality is a human tragedy that could be averted through widespread provision of emergency obstetric and neonatal care, skilled birth attendance, and increased use of contraceptive methods [32,33]. While interventions targeting safer pregnancy and childbirth have been evidenced in many resource-poor countries including Ethiopia [17], 90% of women delivered at home in 2011 [3]. Eliminating inequalities in women’s access to fully equipped facilities [5,6], and improving women’s perceptions of facility delivery through the promotion of respectful maternity care, for example [34-36], are imperative to closing the gap in excess mortality globally among poor women of reproductive age. Until the value of women as irreplaceable pillars of families and communities is recognized, we will continue to see the devastating ripple effects of their deaths play out in the lives of their children.

## Competing interests

The authors of this paper have no conflicts of interest to report.

## Authors’ contributions

MM: Participated study design, coordinated the study, analyzed the data and drafted the paper. AY: Designed the study, conducted interviews, analyzed the data, and structured the paper. IM: Coordinated the fieldwork, analyzed the study and commented on the draft. AW: Participated in study design and coordination. All authors read and approved the final manuscript.

## Peer review

Reviewer reports for this article can be found in Additional file 1.

## Supplementary Material

Additional file 1 Click here for file

## Declarations

This article has been published as part of *Reproductive Health* Volume 12 Supplement 1, 2015: True costs of maternal death. The full contents of the supplement are available online at http://www.reproductive-health-journal.com/supplements/12/S1. Publication charges for this supplement were funded by Family Care International and the FXB Center for Health and Human Rights.
