# Supplementary material for: Impacts of maternal mortality on living children and families: A qualitative study from Butajira, Ethiopia
Source: Reprod Health. 2015 May 6;12(Suppl 1):S6. doi: 10.1186/1742-4755-12-S1-S6 (PMC4423766; doi:10.1186/1742-4755-12-S1-S6)
Supplement: Additional file 1 [file 1742-4755-12-S1-S6-S1.pdf]

Referee's comments to the authors– this sheet WILL be seen by the author(s) and published with the article

Title	Impacts of maternal mortality on living children and families: A qualitative study from Butajira, Ethiopia
Author(s)	Mitike Molla, Israel Mitiku, Alemayehu Worku, Alicia Ely Yamin
Referee's name	Solomon Tessema Memirie

When assessing the work, please consider the following points, where applicable:

1. **Is the question posed by the authors new and well defined?**
2. **Are the methods appropriate and well described, and are sufficient details provided to replicate the work?**
3. **Are the data sound and well controlled?**
4. **Does the manuscript adhere to the relevant standards for reporting and data deposition?**
5. **Are the discussion and conclusions well balanced and adequately supported by the data?**
6. **Do the title and abstract accurately convey what has been found?**
7. **Is the writing acceptable?**

Please make your report as constructive and detailed as possible in your comments so that authors have the opportunity to overcome any serious deficiencies that you find and please also divide your comments into the following categories:

- Major Compulsory Revisions (which the author must respond to before a decision on publication can be reached)
- Minor Essential Revisions (such as missing labels on figures, or the wrong use of a term, which the author can be trusted to correct)
- Discretionary Revisions (which are recommendations for improvement but which the author can choose to ignore)

Where possible please supply references to substantiate your comments.

When referring to the manuscript please provide specific page and paragraph citations where appropriate.

General comments:
No revisions needed.

Major compulsory revisions:

Minor essential revisions:

Discretionary revisions:

(continue on the next sheet)

Referee's comments to the authors– this sheet WILL be seen by the author(s) and published with the article

Title	Impacts of maternal mortality on living children and families: A qualitative study from Butajira, Ethiopia
Author(s)	Mitike Molla, Israel Mitiku, Alemayehu Worku, Alicia Ely Yamin
Referee's name	Diane Cooper

When assessing the work, please consider the following points, where applicable:

1. Is the question posed by the authors new and well defined?
2. Are the methods appropriate and well described, and are sufficient details provided to replicate the work?
3. Are the data sound and well controlled?
4. Does the manuscript adhere to the relevant standards for reporting and data deposition?
5. Are the discussion and conclusions well balanced and adequately supported by the data?
6. Do the title and abstract accurately convey what has been found?
7. Is the writing acceptable?

Please make your report as constructive and detailed as possible in your comments so that authors have the opportunity to overcome any serious deficiencies that you find and please also divide your comments into the following categories:

- Major Compulsory Revisions (which the author must respond to before a decision on publication can be reached)
- Minor Essential Revisions (such as missing labels on figures, or the wrong use of a term, which the author can be trusted to correct)
- Discretionary Revisions (which are recommendations for improvement but which the author can choose to ignore)

Where possible please supply references to substantiate your comments.

When referring to the manuscript please provide specific page and paragraph citations where appropriate.

General comments: This is a very interesting article examining the effects of a mother's death during childbirth on children. The question is well defined. The methods appropriate to the study. The data is interesting and the evidence well presented. The manuscript adheres to standards for reporting. The article presents novel and very worthwhile findings. I have a few minor comments on the discussion and conclusion (see below)

Major compulsory revisions:

Minor essential revisions: I would have like to see some comparison in the discussion between these findings and the findings elsewhere. For example, in the results section, the authors mention that unlike in Malawi, South Africa and Tanzania, there did not appear to be procedures or a common practice of care and placement of children with other family members following maternal death. The authors could expand on this and the implications comparatively. Did the author probe for this and what the practice is? Why it is the case that this is not the case? This should be picked up in the discussion. In the conclusion, it would be good to see a few recommendations for how maternal mortality in Ethiopia could be decreased – what interventions are necessary. While this is being implemented, can the authors recommend any steps to alleviate the negative impact of maternal deaths on children?

Discretionary revisions:

Supplement Editors' comments

Please authors: regarding references not coming from Journals, please provide link and the date where this link has been accessed.
